# Food Safety Evaluation of Potential Human Exposure to Toxic Metals in Tuna Species

**DOI:** 10.3390/jox16040150

**Published:** 2026-08-14

**Authors:** József Lehel, Mihály Németh, Péter Palotás, Balázs Berlinger

**Affiliations:** 1 Department of Food Hygiene, Institute of Food Chain Science, University of Veterinary Medicine Budapest, István u. 2, H-1400 Budapest, Hungary; nemeth.mihaly@hotmail.com; 2 Department of Refrigeration and Livestock Products Technology, Hungarian University of Agriculture and Life Science, H-1118 Budapest, Hungary; peter.palotas72@gmail.com; 3 Department of Animal Hygiene, Herd Health and Mobile Clinic, University of Veterinary Medicine Budapest, István u. 2, H-1078 Budapest, Hungary; berlinger.balazs@univet.hu

**Keywords:** potentially toxic elements, yellowfin tuna, bluefin tuna, food safety, consumer safety, estimated daily intake, target hazard quotient, hazard index

## Abstract

Fish has secured its spot as an important source of human nutrition with its extraordinarily rich nutritional contents; however, it also has the ability to bioaccumulate different potentially toxic elements. They can then end up in the human diet, inducing a wide range of negative effects on consumers’ health. The aim of this study was to measure and evaluate arsenic (As), cadmium (Cd), mercury (Hg), and lead (Pb) concentrations in Yellowfin tuna (*Thunnus albacares*) and Bluefin tuna (*Thunnus thynnus*) acquired at a Hungarian fishery market. The metal concentrations in the flesh of the investigated fish were measured by the ICP-MS method. The average concentrations of metals were As: 2.23, Cd: 0.01, Hg: 0.22, and Pb: 0.29 mg/kg in Yellowfin tuna, and those in Bluefin tuna were As: 1.26, Cd: <0.004, Hg: 0.47, and Pb: 0.28 mg/kg. Pb was found to be over the limit set by the European Commission of 0.3 mg/kg in 17.5% of samples of both Yellowfin and Bluefin tuna. Based on the calculated EDI values, As and Cd content was acceptable, but Hg and Pb were non-compliant. THQ was >1 for Hg in Bluefin tuna and for Pb in both tuna species. HI was >1 for the mixture of metals for adults and children in both tunas. These results indicate potential health risks for consumers over long-term consumption of these fish.

## 1. Introduction

The popularity of seafood and, more specifically, fish, has grown over the past decades at a considerable rate. This can be attributed to multiple factors such as leaps in fishing and fish farming technology allowing for the harvest of larger quantities of fish and the development of transportation, which allows for fish to be transported to any part of the world without going bad. Another big factor is globalization, allowing information, culture, and food to spread and inspire new parts of the world, such as ready-to-eat food products, e.g., sushi and sashimi, which have proven to be widely accepted and loved delicacies around the globe. Furthermore, globalization due to rapid population growth and the resulting need for urbanization requires large-scale farming and an increased rate of production of all sorts of products [1,2,3].

The notion of fish being a healthy part of the diet has also considerably boosted the demand for fish products in the market. This is true to an extent since fish is nutrient dense, packed with omega-3 fatty acids, fats, amino acids, vitamins, and very high-quality proteins and minerals [1,4,5,6]. This is further boosted by the National Fisheries Institute (NFI) recommending the consumption of two servings of fish per week and studies proving the health benefits [7].

While fish are packed with beneficial nutrients, they may also contain and accumulate potentially toxic elements and compounds that are harmful to consumers at elevated doses. The higher the fish is in the food chain of the ecosystem, the greater the bioaccumulation of toxic metals in the tissues [8].

Potentially toxic metals can be found in the marine environment in different forms and states, such as in organic or inorganic compounds. Their concentration in aquatic environments can be influenced by various factors. These metals find their way into the marine ecosystem through various pathways. These include physical or chemical erosion and volcanic eruptions, which release metals from their natural sources into the soils and surface waters, or anthropogenic sources such as wastewater from different industries like electronics, metal finishing and electroplating, or leaches from landfills, mining, urban runoff or municipal wastewaters. The latter has a substantially greater footprint when it comes to the contributions to contamination of waters with these toxic substances [9].

Once these metals enter the water systems they can reach the organisms at all trophic levels through multiple different pathways. These elements can be taken up by the fish through various biological pathways and may accumulate to different levels within their tissues. Fish can be exposed to these elements through contact with the water they live in, water they filter through their gills, and more importantly through their diet. Their diet plays a very important role since biomagnification up the food-chain or web can cause a sharp increase in the concentration of the contaminants in the tissues [9].

Fish can have positive effects on human health if they are not overly exposed to toxins, which are controlled by various food safety laws and measures, and/or are consumed in amounts not exceeding the recommended portions. The long-term accumulation of these elements in the human body can lead to serious health issues (developmental anomalies, nervous system damage, liver and kidney damage, reproductive and hematological disorders, cancer and cardiovascular diseases) [1,10,11].

The toxicity of As has a diverse range of organs and tissues it affects, thus there are multiple clinical signs and diseases that can be observed. These can be skin related such as hyperkeratosis, hyperkeratotic warts and corns at the end of the extremities, and pigmentation defects such as hyper- and hypopigmentation on the face, neck, and back. Being chronically exposed to inorganic arsenic can elicit neurotoxic effects in both the peripheral and central nervous systems. On the other hand, acute toxicosis has a quicker poisoning-like effect. One can observe the onset of acute profuse vomiting, diarrhea, colic, salivation, fever, cardiovascular disturbances and disturbances in the central nervous system which in severe cases may cause death. It also has carcinogenic properties, which mainly attack and cause cancer formation in the lungs, kidneys, bladder and skin [12,13].

Acute toxicity caused by Cd is relatively rare, but in the case of exposure to a high dietary level, one may experience gastrointestinal symptoms. The chronic buildup of Cd in the body can lead to renal dysfunction, cardiovascular problems effects on the bone and even cancer as it is deemed carcinogenic [14]. The toxic effects of Cd are widespread with effects on multiple organs including the kidney, heart, lung, skeletal system, testes, placenta, brain and central nervous system [12,13].

Exposure to Pb over a long period of time or at higher levels can have multiple negative health effects. Poisoning caused by Pb can manifest headaches, irritability, abdominal pain and various nervous system problems. It may also cause hematopoietic and nephrotoxic effects besides developing poor attention spans, epigastric, constipation, vomiting, convulsions, coma and in some cases even death [12,14].

Methyl Mercury (MeHg) poisoning can have multiple toxic effects on human health. MeHg can interfere with protein synthesis, can disturb the function of microtubules and can modify neurotransmission by increasing the intracellular Ca^2+^. MeHg can also attack the nervous system causing various symptoms such as numbness in the hands and feet, coordination difficulties and visual and auditory symptoms. On top of these symptoms, ischemic strokes, dementia and depression can develop as well as toxicity to the kidneys, gastrointestinal issues can arise with ulceration and hemorrhages [12,13]. It is especially toxic to infants. The freshly developing nervous system of newborns is highly sensitive to the neurotoxic impact of organic MeHg. This compound can also be dangerous before the infant is born, as it can pass through the placenta and reach the embryo [12,13].

In wild fish it is virtually impossible to prevent or control the consumption and accumulation of these elements. The best humans can do is to decrease the pollution of the ecosystems while monitoring and setting maximum concentrations for the contaminants in the fish that are sold for human consumption.

To ensure the safety of the people living in the European Union, rules and regulations have been written up intended to prevent dietary exposure to potentially toxic quantities of toxic contaminants in the foodstuffs made available to the public. To prevent the dangerous levels of contaminants in food sold to the consumers, the European Commission has been setting maximum limits on the amounts of environmental and other contaminants that can be present in food brought to market within its member states. Commission Regulation (EU) 2023/915 of 25 April 2023 laid down maximum limit (ML) for metals [15]. Based on this, the ML of inorganic As (iAs) ranges from 0.010 to 0.30 mg/kg for different types of food; however, there is currently no limit value for fish. The regulated official ML for Cd 0.10 mg/kg ww (wet weight) for Tuna species. Generally, the ML for Pb is 0.30 mg/kg and for mercury (Hg) is 1.00 mg/kg in flesh of fish [15].

This study aims to investigate the most important potentially toxic metals, such as As, Cd, Hg and Pb in the edible tissue of the marine fish, and to assess and evaluate their potential risk to human consumers.

## 2. Materials and Methods

### 2.1. Samplings

Forty samples were taken from Bluefin tuna (*Thunnus thynnus*) and 40 specimens were sampled from Yellowfin tuna (*Thunnus albacares*) at a Hungarian fishery market. However, Bluefin tuna was originated from FAO 37.1.1 Fishing area (Mediterranean and Black Sea (37), Western Mediterranean (Subarea 37.1), Balearic (Division 37.1.1), and Yellowfin tuna from FAO 57 Fishing area (Indian Ocean, Bay of Bengal (57.1), Sri Lanka) [16,17].

### 2.2. Methods

#### 2.2.1. Reagents and Analytical Standards

Unless otherwise stated, all chemicals used for sample preparation were of trace analytical grade. Nitric acid (69%, Aristar), hydrochloric acid (37%, Aristar), hydrogen peroxide (30%, AnalaR NORMAPUR), and ethanol (AnalaR NORMAPUR) were obtained from VWR International Ltd. (Leicestershire, UK). Ultrapure water (18.2 MΩ·cm) was produced using a Purite Select Fusion 160 BP water purification system (SUEZ Water Technologies & Solutions, Trevose, PA, USA).

External calibration of the ICP-MS instrument was performed using Instrument Calibration Standard 2 (TruQms), while Instrument Internal Standard Mix (TruQms) was used for internal standardization. Both solutions were supplied by PerkinElmer Inc. (Waltham, MA, USA). The certified reference material ERM-CE278k (mussel tissue; Joint Research Centre, Geel, Belgium) was used for quality control measurements. Argon gas (purity 4.8) was supplied by Messer Hungarogáz Ltd. (Budapest, Hungary).

#### 2.2.2. Sample Preparation

Approximately 0.5 g of homogenized fish tissue was accurately weighed into CEM MARS XPress Teflon (CEM Corporation, Matthews, NC, USA) digestion vessels. Each sample was mixed with 5 mL of concentrated nitric acid and 5 mL of hydrogen peroxide before microwave-assisted digestion using a CEM MARS6 system (CEM Corporation, Matthews, NC, USA). The digestion program consisted of a 35 min temperature ramp to 200 °C, followed by a 50 min holding period at this temperature. Microwave power was set to 1700 W, and up to 40 vessels were processed simultaneously.

After digestion, the solutions were quantitatively transferred into 50 mL polypropylene tubes (Deltalab, Rubí, Barcelona, Spain). The digests were diluted to a final volume of 25 mL with ultrapure water. Prior to ICP-MS analysis, each digest was diluted five-fold in 12 mL polypropylene tubes (Deltalab, Rubí, Barcelona, Spain). The diluted solutions contained 0.4 mL ethanol and 100 µL internal standard solution, providing final internal standard concentrations of 1 µg/mL for bismuth (Bi), germanium (Ge) and indium (In).

Quality control samples and procedural blanks were prepared following the same protocol as the analytical samples. Between digestion batches, the Teflon vessels were cleaned using 0.15 M hydrochloric acid.

#### 2.2.3. Instrumentation

Elemental analysis was performed using a NexION 2000 inductively coupled plasma mass spectrometer (PerkinElmer, Waltham, MA, USA) operated in helium kinetic energy discrimination (KED) mode. The low and high helium cell gas flow rates were set to 5.6 and 6.7 mL/min, respectively.

The ICP-MS operating conditions were as follows: RF power, 1600 W; free-running 34.5 MHz solid-state RF generator equipped with a LumiCoil; plasma gas flow rate, 15 L/min; auxiliary gas flow rate, 1.2 L/min; nebulizer gas flow rate, 1.03 L/min; and a low-volume quartz Meinhard type C nebulizer.

#### 2.2.4. Validation of the Analytical Method

The analytical procedure, including the sample preparation and the ICP-MS determination, was validated in accordance with the performance criteria described in Commission Decision 2002/657/EC and the Eurachem Guide [18,19].

Limits of detection (LOD) and limits of quantification (LOQ) were estimated as three and ten times the standard deviation of the procedural blank concentrations, respectively. Method performance was evaluated by analyzing ten replicates of the certified reference material ERM-CE278k. Precision was expressed as the relative standard deviation (RSD, %), trueness as the relative bias (%), and accuracy as the percentage recovery (%) calculated from the certified reference values.

Calibration linearity was assessed using the corresponding calibration functions, yielding correlation coefficients (r) of at least 0.999 for all monitored isotopes. Matrix effects were not evaluated independently because signal variations were compensated by continuous internal standardization applied to all calibration standards and samples.

The monitored isotopes together with their corresponding LOD, LOQ, precision, trueness, and recovery values are summarized in Table 1. Acceptance criteria were defined as a precision not exceeding 15% RSD and trueness within ±20%. The recoveries obtained for the certified reference material ranged from 99% to 110% for all analytes investigated.

### 2.3. Statistical Analysis

The concentrations of As, Cd, Hg and Pd in Yellowfin tuna and Bluefin tuna were compared using a *t*-test. The statistical analysis was conducted using the Excel program. For the metals that had samples with concentrations below the LOD, the *t*-test was done twice, taking the values to be the LOD and as 0 to test for differences.

The distribution of the results was normal. The *t*-test shows if statistically there is a difference between the two groups of data. If the answer is ≤0.05 it indicates a difference in the two sets of data, while a value > 0.05 indicates that the sets of data are statistically the same. For As, Cd and Hg the test deduced that the concentrations were different. Pb was the only metal that was tested where the samples showed no statistical difference in concentrations between the two fish species.

### 2.4. Calculation of Exposure

The Joint FAO/WHO Expert Committee on Food Additives (JECFA) has set a maximum amount of potentially toxic metals found in food. This is to remove products from the market, which can induce damage to health over a long-life uptake, in order to protect the consumers’ health. These limits are called the Provisional Tolerable Daily, Weekly and or Monthly Intake (PTDI, PTWI and PTMI). Since some of these have since been withdrawn the estimated daily intake (EDI) was calculated for the metals investigated [20,21,22,23,24,25].

These calculations were conducted using data of average annual fish intake in the European Union. According to the European Food Safety Association (EFSA) this is 23.97 kg/year/person, which correlates to 65.7 g/day/person of fish [26].

The EDI calculations were done by multiplying the heavy metal concentrations (C, mg/kg) in the samples investigated with the average daily fish consumption of 65.7 g/day/person (Cons, g/day) [25,26], which was then divided by an average human body weight of 70 kg (Bw, kg).

EDI (mg/kg) = (C × Cons)/Bw(1)

A further calculation to assess the safety/toxicity of different concentrations of the potentially toxic materials in foods is the Target Hazard Quotient (THQ) suggested by the US-Environmental Protection Agency (US-EPA). The THQ was developed to evaluate the probability of the adverse health effects of the potentially toxic trace elements found in the foods, on the people exposed to them through their diet. THQ describes the non-cancer risk of the contaminants by dividing exposure dose (i.e., dietary intake, EDI, mg/day) by the reference dose (RfD, mg/day) [27].

THQ (non-dimensional) = EDI/RfD(2)

If THQ is <1, it indicates that the hazardous metal is not at a harmful concentration, while if THQ is >1, the concentration of the hazardous metal is potentially harmful [27].

Using the target hazard quotients for the metals found in the fish, further assessment was performed to calculate the health risks of the mixture of metals. For this Hazard Index (HI) calculations were used with the following formula, where a, b, c… indicates the metal that the THQ applies to [28]:

HI (non-dimensional) = THQa + THQb + THQc …(3)

HI being <1.0 indicates a low chance for adverse effects by the mixture of metals, while HI being >10 corresponds with greater risk of adverse health effects [28,29].

## 3. Results and Discussions

### 3.1. Concentration of Metals

The concentrations of the potentially toxic metals (As, Cd, Hg, Pb) investigated in the flesh of Yellowfin tuna and Bluefin tuna are summarized in Table 2.

As can be seen in Table 2, Pb was the only one above the ML (0.3 mg/kg) set by the European Commission in 17.5% of the samples investigated from both tuna species [15].

The concentrations of As, Cd and Hg all stayed within the Official Limits set by the European Commission, while the concentration of Pb reached levels greater than the officially permitted ones by the European Commission. In the case of As there is no official limit set for the maximum concentration in tuna, while in case of Cd it is 0.1 mg/kg, in Hg it is 1 mg/kg and in Pb it is 0.3 mg/kg.

When doing the *t*-test for As, Cd, Hg and Pb it showed that the concentrations were different for As, Cd and Hg, while Pb showed no statistical difference in the concentrations between the two fish species. In the case of Cd and Pb we did the *t*-test twice. Once taking the values smaller than the LOD, as the LOD value and once as 0. The difference it made was not relevant enough to change the statistical outcome.

Basically, the Pb was the only metal that reached levels greater than the officially permitted ones by the European Commission and showed statistical relevance, but EDI and THQ values were calculated for all investigated metals.

The pollution and the contamination of the waterbodies is highly affected by natural, geographical and anthropogenic factors, resulting in high variability. From this study we could deduce that the As, Cd and Hg levels were most likely different between the two regions, namely FAO 57, 57.1 and FAO 37, 37.1 and 37.1.1, while statistically the Pb concentrations were the same in both species hinting at the fact, that the surroundings had similar levels of Pb pollution. These speculations are backed by the *t*-test performed, using the data collected from the two fish species. The *p*-values obtained for As, Cd and Hg were all well < 0.001, indicating statistically significant differences between the two tuna species. In contrast, the *p*-value for Pb was 0.215, indicating no statistically significant difference.

Generally, the contamination levels are not constant even within a fishery area. But as a rule of thumb, one can expect the levels of contamination to be higher the closer it is to a densely populated area where there are agriculture and industry [30]. This can be seen in a study that examined the level of metal contaminants in water at different locations, for example the Coastal Bay of Bengal and Offshore Bay of Bengal, where the Pb concentrations were 0.72 μg/L and 0.49 μg/L, respectively. Similar tendencies were shown for the other metals tested in the water and plankton. For plankton respectively to the region, 21.1 μg/g and 16.2 μg/g of Pb were found [31]. The concentrations can also be highly influenced by external factors other than the number of contaminants flowing into the water body from the source. Studies have shown, the power of tidal courants around the coast of India influencing the concentration of heavy metals in the water. Where the tidal movement transports heavy metal settled in the sediment away from the initial site of pollution diluting the concentrations [31,32]. Another aspect investigated was the effect of the different seasons in India namely the monsoon season and the elevating effect it had on the heavy metal concentrations in the water. The study found the highest contamination level during the monsoon followed by the pre-monsoon and the post-monsoon periods using multivariate pollution indices from 9 different regions. One of the indices was the ecological risk index which ranged from 0.046 to 115.71 post-monsoon, 0.11–120.62 during monsoon and 0.11–103.94 pre-monsoon. A further index used was the Potential ecological risk which ranged from 81.37 to 138.03 post-monsoon, 76.89–143.36 in monsoon and 79.70–124.13 in pre-monsoon. Both indices showed that the highest risk of heavy metals was found during the monsoon [32].

Geographic characteristics of the waterbodies also play a role in their ability to accumulate and concentrate on environmental metal pollutants. This was found to be the case for the Mediterranean Sea as it is a semi-enclosed basin, which has a vast coastline that has been heavily urbanized. This means that there is a vast source of potential pollution but a limited passage of escape/clearing of the heavy metal pollution resulting in their retention. A study conducted on the coast of Libya investigated the heavy metals in the water in Susah and Tobruk. In Susah they found a mean concentration of 623 μg/L for Cd and 79.8 μg/L for Pb while in Tobruk 121 μg/L of Cd and 34.07 μg/L of Pb [33] were detected.

A further aspect that has been investigated around the coast of the Bay of Bengal is the properties of the sediment and the different retention abilities that they have, depending on the composition and the grain size. It was found that different elements like Fe, Cd, Cr, Ni, Pb and Zn are more concentrated in muddier, silty clay sediments with higher organic matter which are usually found in deeper waters, whereas Co and Cu are found in more sandy sediments which are in shallower regions. In this study off the Coromandel Coast of India the concentration of 3.30–19.80 mg/kg of Cd was found and 2.67–49.63 mg/kg of Pd was determined in the surface sediment [34]. One could expect waters with the respective sediments to have a larger concentration of the specific heavy metals in return also influencing the contamination of the fish with said heavy metals over their lifetime.

The concentrations of Pb in this investigation were quite a bit higher than ones found in other scientific literature. Our concentrations of Pb averaging a 0.29 ± 0.23 mg/kg in Yellowfin tuna and 0.28 ± 0.22 mg/kg in Bluefin tuna, while investigations in Galle, Mutwal, Negombo and Trincomalee areas of Sri Lanka reported concentrations of 0.09 ± 0.12 mg/kg ww [35], while another study from Sri Lanka reported similar concentrations in Yellowfin tuna at 0.06 ± 0.06 mg/kg ww [20]. A further study carried out in the north-east of Brazil yielded once more a similar result of 0.09 ± 0.06 mg/kg ww [36]. Continuing the pattern a study on various tuna species (Skipjack [*Katsuwonus pelamis*], Yellowfin tuna, Bigeye tuna [*Thunnus obesus*]) in Ecuador once again reported concentrations of 0.06 ± 0.05 mg/kg ww [37].

The concentrations of total As in our study were averaged a value of 1.26 ± 0.63 mg/kg in the Bluefin tuna and 2.23 ± 0.12 mg/kg in Yellowfin tuna. These values fall within the range of concentrations that are reported in international papers. A paper investigating tuna species in Aracaju, Sergipe, north-eastern Brazil reported levels of 1.30 ± 0.34 mg/kg As [36], while a paper investigating tuna from Japanese restaurants in the Republic of Korea reported findings of 0.98 ± 0.47 mg/kg similarly within our range [38].

However, one can find literature reporting concentrations higher than ours such as the paper which investigated Yellowfin tuna in Northwest Spain, with concentrations of 3.78 ± 2.24 mg/kg [39], which was close to the concentrations found in Indonesian Yellowfin tuna in Jakarta at 3.47 ± 0.21 mg/kg [40]. Papers have also reported cases where the As load of the fish tested was lower than what we found. Bluefin tuna from the Canary Islands in Spain were found to have lower As concentration at levels ranging from 0.02 to 0.59 mg/kg [41].

The concentrations of Cd in our samples had an average concentration of <0.004 mg/kg in the Bluefin tuna and 0.01 ± 0.002 mg/kg in the Yellowfin tuna. The only paper that reported values similarly low as the ones found in this paper was one reporting on Mediterranean meals with a mixture of fish species and further components of the diet at concentrations ranging from 0.0006 to 0.005 mg/kg [27].

Further reports reported substantially higher values for Cd in fish. Sri Lankan Yellowfin tuna was found to have Cd concentration between 0.01 ± 0.01 mg/kg and 0.02 ± 0.02 mg/kg [35,42] and Trincomaleean Yellowfin tuna reported to have Cd levels ranging from <0.01 to 0.13 mg/kg [43].

Ormaza-Gonzáles et al. (2020) reported on the Cd concentrations in different tuna species (Skipjack, Yellowfin tuna, Bigeye tuna) from Ecuador, with even higher concentrations averaging 0.03 ± 0.03 mg/kg [37]. The paper reporting the highest concentrations of Cd was testing tuna flesh from north-eastern Brazil with a concentration of 0.08 ± 0.02 mg/kg [36].

The average concentrations for Hg investigated in the Bluefin tuna in this paper was 0.47 ± 0.04 mg/kg while that of the Yellowfin tuna was 0.22 ± 0.02 mg/kg. Other scientific research has found similar results to ours. One examined Yellowfin tuna in Sri Lanka finding concentrations of 0.30 ± 0.14 mg/kg [35] while another paper examining the belly flesh of Yellowfin tuna in Sri Lanka found concentrations of 0.39 ± 0.19 mg/kg [42].

There is also plenty of research showing more elevated levels of mercury in tuna. Ruales-Inzunza et al. (2018) reported concentrations of 0.71 ± 0.62 mg/kg of Hg in Yellowfin tuna around the Pacific Ocean around Mexico [44]. Similar Hg levels of 0.68 ± 0.08 mg/kg were found in the flesh of Yellowfin tuna caught in Jakarta, Indonesia [40]. Further research on Yellowfin tuna and Skipjack tuna from Foggia, Italy reported values of 0.55 ± 0.43 mg/kg and 0.40 ± 0.31 mg/kg [45].

### 3.2. Potential Exposure

The non-carcinogenic health risk associated with dietary exposure to the investigated toxic elements was assessed using the Target Hazard Quotient (THQ) and Hazard Index (HI) approach based on Reference doses (RfDs), which is widely applied for screening-level human health risk assessment.

The calculated EDI values are presented in Table 3, which shows the estimated quantity of potentially toxic metals that a European person consumes with fish on average per day per kg bodyweight. Then they were compared the relevant RfD values such as 0.3 μg/kg/day for As [21,46], 1.0 μg/kg/day for Cd [22,46], 0.3 μg/kg/day for Hg [23,47] and 0.16 (adult) and 0.26 (children) μg/kg/day for Pb [24,46]. Based on these calculated values, it can be stated that these fish do not induce health risk over a long-life uptake.

Most of As, about 95%, can be found in the form of organic derivatives which are relative non-toxic and can be excreted from the human body. Generally, based on published literature, inorganic arsenic (iAs) generally represents only a small proportion (commonly assumed to be approximately 5%) of the total arsenic present in marine fish. Therefore, in the absence of arsenic speciation data, the potential exposure to iAs was estimated using this commonly applied assumption [21]. Considering this scientific information, only the potential exposure to iAs was calculated.

The calculated EDI values for iAs and Cd were non-compliant in Bluefin and Yellowfin tuna, and for Hg in Yellowfin tuna. However, the EDI value of Hg in Bluefin tuna was 1.47-times higher compared to RfD, and that of the Pb were 1.64-times (adult) and 1.01-times (children) higher than the reference dose in Bluefin tuna, and 1.7-times (adult) and 1.05-times higher in Yellowfin tuna.

Based on these calculated values, it can be stated that these fish can induce health risk over a long-life uptake due to the calculated EDI values for Hg in Bluefin tuna, and for Pb in both tuna species investigated to adult people and children.

**Figure 1:**
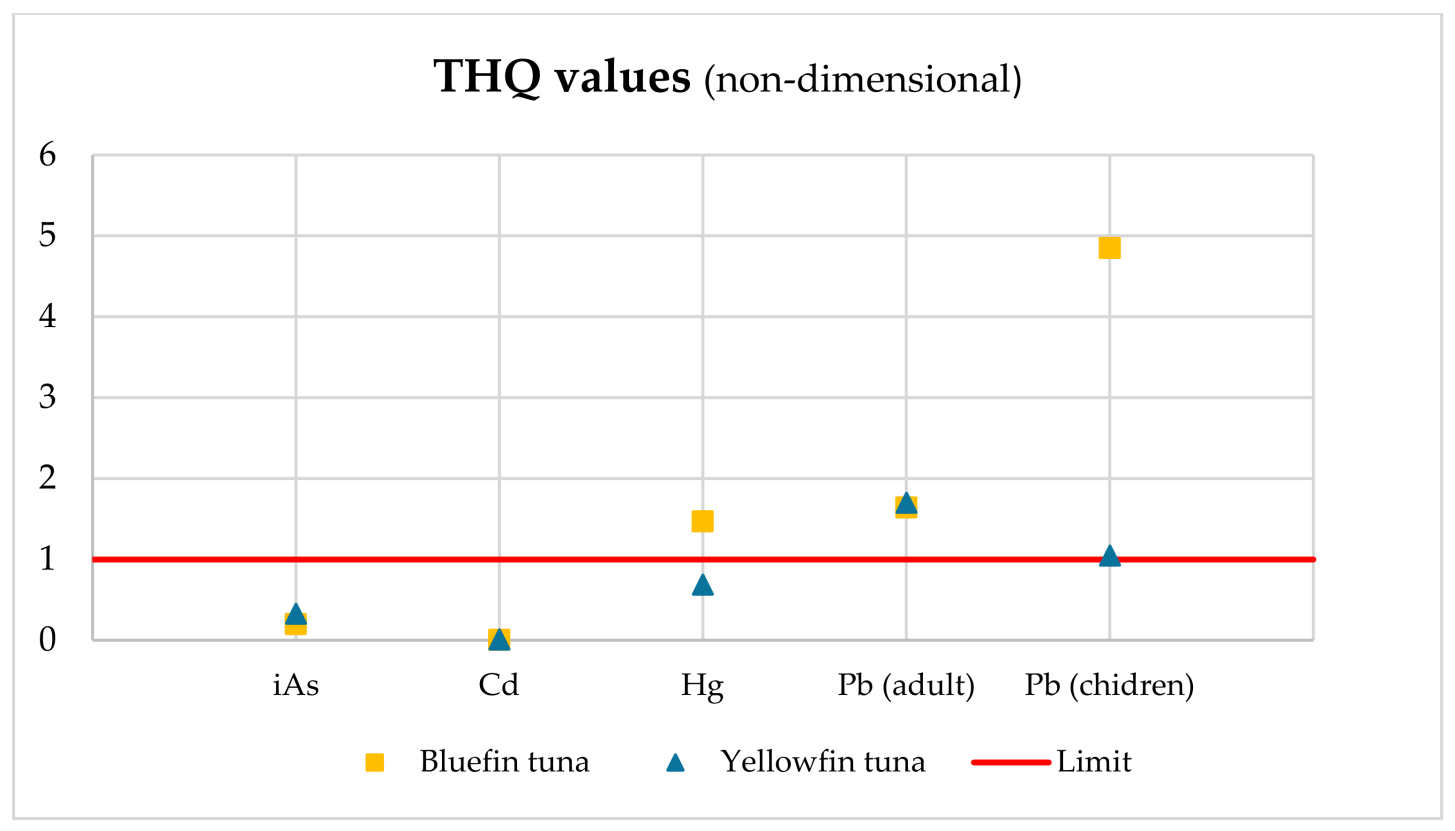
THQ values (non-dimensional) for the potentially toxic metals investigated.

The calculated THQ values were <1 for iAs, Cd, but >1 for Hg in Bluefin tuna and for Pb in Bluefin and Yellowfin tuna (Figure 1). It means that the concentrations of Hg and Pb are potentially harmful to the consumers during long-life uptake in the fish that were tested [25].

HI values demonstrate that the chances for health risks and adverse effects by the mixture of metals caused by both fish species are greater since they were over 1.0 (Bluefin tuna: 3.314 for adult, and 6.52 for children; Yellowfin tuna: 2.73 for adult, and 2.08 for children) [26,27].

Basically, there are lots of factors which can influence the heavy metal content of the marine ecosystems. It may be difficult to pinpoint direct correlations between the heavy metal concentrations in fish and their surroundings, since the bioaccumulation takes place over a long period of time during which the fish cover a large area which may have largely varying levels of contamination. This can be seen in the variability of the measured concentrations within the 40 specimens examined in both species of tuna.

Our results refer only to the investigated catches; however, they can give general view about the potentially toxic metal burden on the given fishing areas, and thus to the consumers. Certainly, it would have been beneficial to examine the pollution levels of the given fishing areas as well, which would have added to the current scientific knowledge, but we were unable to carry out this sampling and analysis.

The present risk assessment represents a screening-level evaluation based on average fish consumption and the analytical approach described above. Alternative exposure scenarios (e.g., species-specific consumption data or lower-bound/upper-bound treatment of left-censored data) may result in different exposure estimates and should be considered in future studies.

In summary, these results should be interpreted as identifying consumption scenarios where screening-level, non-carcinogenic concerns may arise, rather than as evidence of adverse health effects at the population level. The findings point to the need for continued monitoring of inorganic arsenic, cadmium, and lead in bluefin and yellowfin tuna species marketed for human consumption and underscore the importance of risk-based communication over broad, population-level advice. Future studies should validate these screening-level findings using species-specific consumption data, direct inorganic arsenic speciation, and more sensitive mercury analysis methods, thereby enabling more robust regulatory and consumer risk assessments.

## 4. Conclusions

In conclusion, this study on Yellowfin tuna (from FAO 57: Indian Ocean, Bay of Bengal (57.1), Sri Lanka) and Bluefin tuna (from FAO 37.1.1, Mediterranean and Black Sea (37), Western Mediterranean (Subarea 37.1), Balearic (Division 37.1.1)) found, that from metals examined (As, Cd, Hg and Pb) that could possibly pose a risk to the human health at higher concentrations, only one qualified to have concerns about. The concern is present if individual fish are examined that belong to the 7 out of 40 which had higher than the permitted Pb levels in them in both Yellow and Bluefin tuna. While if one looks at bulk values the average concentration in both cases falls below the regulated official maximum levels of Pb concentration, like in the case of the other heavy metals.

During the evaluation the grand picture has been looked at, and the average concentration has been used for everyday consumption. Even though the outliers are compensated for, it does not mean that they should be disregarded completely. They can be used for long-term studies on the contamination of fish and the fishing areas.

The average concentration values were used to conduct further calculations to determine the potential exposure in terms of EDI, THQ and HI. The EDI values exceeded the reference doses for Pb to both adult people and children in both tested tuna species, and for Hg in Bluefin tuna. The Target Hazard Quotient values indicate that the concentrations of As and Cd are non-objectionable; however, the concentrations of Hg in Bluefin tuna, and that of Pb in both tuna species are potentially harmful for adults and children. The Hazard Index of the mixture of the metals were >1 for both Yellowfin and Bluefin tuna posing greater risk for potential adverse effects from either one. Based on the calculated EDI, THQ, and HI values, these fish can induce potential health risk for human consumers over a long-life consumption.

## Figures and Tables

**Table 1:** Analytical performance characteristics of the ICP-MS method, including monitored isotopes, limits of detection (LOD), limits of quantification (LOQ), precision, trueness, and recovery values determined using the certified reference material ERM-CE278k.

Element	Isotope (*m*/*z*)	LOD (mg/kg)	LOQ (mg/kg)	Precision %	Trueness %	QC (ERM-CE278k)		
						Certified Value (mg/kg)	Measured Value (mg/kg)	Recovery (%)
**Arsenic (As)**	75	0.005	0.017	3.9	7.4–15.4	6.7 ± 0.4	7.4 ± 0.3	110
**Cadmium (Cd)**	111	0.004	0.013	2.4	5.5–10.5	0.336 ± 0.025	0.361 ± 0.09	108
**Mercury (Hg)**	201	0.005	0.017	6.4	0.4–14.2	0.071 ± 0.007	0.078 ± 0.005	110
**Lead (Pb)**	207	0.05	0.17	8.5	−6.7–8.8	2.18 ± 0.18	2.16 ± 0.18	99

**Table 2:** Concentration of heavy metals in the investigated tuna species (mg/kg).

	As	Cd	Hg	Pb
**Yellowfin tuna**				
Average	2.23	0.01	0.22	0.29
±SD	0.12	0.002	0.02	0.23
LOD	0.005	0.004	0.005	0.05
Ratio above LOD (%)	100	55	100	47.5
Official limit	NL	0.10	1.00	0.30
Ratio above ML (%)	NL	0	0	17.5
**Bluefin tuna**				
Average	1.26	<0.004	0.47	0.28
±SD	0.63	0.00	0.04	0.22
LOD	0.005	0.004	0.005	0.05
Ratio above LOD (%)	100	0	100	35
Official limit	NL	0.10	1.00	0.30
Ratio above ML (%)	NL	0	0	17.5

**Table 3:** EDI (µg/kg/day) for the potentially toxic metals investigated.

Metal	EDI Value	
	Bluefin Tuna	Yellowfin Tuna
**As (inorganic)**	0.06	0.10
**Cd**	0.004	0.009
**Hg**	0.441	0.207
**Pb**	0.263	0.272

## Data Availability

The original contributions presented in this study are included in the article. Further inquiries can be directed to the corresponding author.

## References

[1] Plachy M., Bartha A., Budai P., Palotás P., Lehel J. (2022). Toxic elements in Sardina pilchardus and food toxicological significance. Food Addit. Contam. Part B.

[2] Zhou J., Salvador S.M., Liu Y.P., Sequeria M. (2001). Heavy metals in the tissues of common dolphins (*Delphinus delphis*) stranded on the Portuguese coast. Sci. Total Environ..

[3] Türkmen M., Türkmen A., Tepe Y., Ates A., Gökkus K. (2008). Determination of metal, contaminations in sea from Marmar, Aegean and Mediterranean seas: Twelve fish species. Food Chem..

[4] Kinsella J.E., Lokesh B., Stone R.A. (1990). Dietary n-3 polyunsaturated fatty acids in amelioration of cardiovascular disease: Possible mechanisms. Am. J. Clin. Nutr..

[5] Oomen C.M., Feskens E.J., Räsänen L., Fidanza F., Nissinen A.M., Menotti A., Kok F.J., Kromhout D. (2000). Fish consumption and coronary heart disease mortality in Finland, Italy and The Netherlands. Am. J. Epidemiol..

[6] Kris-Etherton P.M., Harris W.S., Apple L.J. (2002). Fish consumption, fish oil, Omega-3 fatty acids, and cardiovascular disease. Circulation.

[7] Lehel J., Yaucat-Guendi R., Darnay L., Palotás P., Laczay P. (2021). Possible food safety hazards of ready-to-eat raw fish containing product (sushi, sashimi). Crit. Rev. Food Sci. Nutr..

[8] Mann R.M., Vijver M.G., Peijnenburg W.J.G.M., Sánchez-Bayo F., van den Brink P.J., Mann R.M. (2011). Metals and Metalloids in Terrestrial Systems: Bioaccumulation, Biomagnification and Subsequent Adverse Effects. Ecological Impacts of Toxic Chemicals.

[9] Saidon N.B., Szabó R., Budai P., Lehel J. (2024). Trophic transfer and biomagnification potential of environmental contaminants (heavy metals) in aquatic ecosystems. Environ. Pollut..

[10] Rajizadeh A.M., Pourbabaki R., Cordaro M., Di Paola R., Fusco R. (2024). Oxidative Stress and Exposure to Metals. Biochemical and Physiological Response During Oxidative Stress—From Invertebrates to Vertebrates.

[11] Varol M., Kaya G.K., Alp S.A., Sünbül M.R. (2018). Trace metal levels in rainbow trout (*Oncorhynchus mykiss*) cultured in net cages in a reservoir and evaluation of human health risks from consumption. Biol. Trace Elem. Res..

[12] Castro-González M.I., Méndez-Armenta M. (2008). Heavy metals: Implications associated to fish consumption. Environ. Toxicol. Pharmacol..

[13] Lehel J., Pleva D., Nagy A.L., Süth M., Kocsner T. (2025). Potential metal contaminations in foods of animal origin—Food safety aspects. Appl. Sci..

[14] Schrenk D., Cartus A. (2017). Chemical Contaminants and Residues in Food.

[15] Commission Regulation (EC) No 2023/915 on Setting Maximum Levels for Certain Contaminants in Food and Repealing Regulation (EC) No 1881/2006. http://data.europa.eu/eli/reg/2023/915/oj.

[16] FAO Fishing Area 37. Mediterranean and Black Sea. https://www.fao.org/fishery/en/area/fao:37/en.

[17] FAO Fishing Area 57. Indian Ocean, Eastern. https://www.fao.org/fishery/en/area/fao:57/en.

[18] Commission Decision No 2002/657 on Implementing Council Directive 96/23/EC Concerning the Performance of Analytical Methods and the Interpretation of Results. http://data.europa.eu/eli/dec/2002/657/oj.

[19] Magnusson B., Örnemark U. (2014). Eurachem Guide: The Fitness for Purpose of Analytical Methods—A Laboratory Guide to Method Validation and Related Topics.

[20] Lehel J., Papp Z., Bartha A., Palotás P., Szabó R., Budai P., Süth M. (2023). Metal Load of Potentially Toxic Elements in Tuna (*Thunnus albacares*)—Food Safety Aspects. Foods.

[21] EFSA (European Food Safety Authority) (2009). Scientific opinion on arsenic in food. EFSA J..

[22] EFSA (European Food Safety Authority) (2009). Scientific opinion of the Panel on Contaminants in the Food Chain on a request from the European Commission on cadmium in food. EFSA J..

[23] EFSA (European Food Safety Authority) (2012). Scientific Opinion on the risk for public health related to the presence of mercury and methylmercury in food. EFSA J..

[24] EFSA (European Food Safety Authority) (2010). Scientific opinion on lead in food. EFSA J..

[25] Chamannejadian A., Sayyad G., Moezzi A., Jahangiri A. (2013). Evaluation of estimated daily intake (EDI) of cadmium and lead for rice (*Oryza sativa* L.) in calcareous soils. Iran. J. Environ. Health Sci. Eng..

[26] European Commission: Food, Farming, Fisheries Oceans and Fisheries: Consumption. https://oceans-and-fisheries.ec.europa.eu/facts-and-figures/facts-and-figures-common-fisheries-policy/consumption_en.

[27] Barreca S., Orecchio S., Orecchio S., Abbate I., Pellerito C. (2023). Macro and micro elements in traditional meals of Mediterranean diet: Determination, estimated intake by population, risk assessment and chemometric analysis. J. Food Comp. Anal..

[28] Lehel J., Plachy M., Palotás P., Bartha A., Budai P. (2024). Possible Metal Burden of Potentially Toxic Elements in Rainbow Trout (*Oncorhynchus mykiss*) on Aquaculture Farm. Fishes.

[29] Djedjibegovic J., Marjanovic A., Tahirovic D., Caklovica K., Turalic A., Lugusic A., Omeragic E., Sober M., Caklovica F. (2020). Heavy metals in commercial fish and seafood products and risk assessment in adult population in Bosnia and Herzegovina. Sci. Rep..

[30] Barbieri M.V., González M.P., Piqué V.A., Blasco J., Eljarrat E. (2025). Contaminants in fish and seafood from the marine environment: A global overview of current status and future perspective. Mar. Pollut. Bull..

[31] Rashid C.P., Jyothibabu R., Arunpadi N., Alok K.T., Vidhya V., Snigtha, Gireeshkumar T.R., Sudheesh V., Marigoudar S.R., Sharma K.V. (2025). Tidal control of heavy metal loading in the nearshore of the northwestern Indian coast. Sci. Total Environ..

[32] Mookan V.P., Machakalai R.K., Srinivasan S., Sigamani S., Kolandhasamy P., Gnanamoorthy P., Moovendhan M., Srinivasan R., Hatamleh A.A., Al-Dosary M.A. (2023). Assessment of metal contaminants along the Bay of Bengal- Multivariate pollution indices. Mar. Poll. Bull..

[33] Maeyouf H., Khattab R.A., Temraz T., Sami M., Ali I., Imanova G. (2025). Heavy metal contamination in seawater, sediments, algae, and fish from Susah and Tobruk, Mediterranean Sea. Water Environ. Res..

[34] Anbuselvan N., Senthil N.D., Sridharan M. (2018). Heavy metal assessment in surface sediments off Coromandel Coast of India: Implication on marine pollution. Mar. Pollut. Bull..

[35] Jinadasa B.K.K.K., Edirisinghe E.M.R.K.B., Ahmad S.B.N. (2013). Level of trace metals Hg, Cd, Pb, Cu, Fe and Zn in yellowfin tuna (*Thunnus albacares*) and swordfish (*Xiphias gladius*) collected from Sri Lanka. J. Nat. Aquat. Resourc. Res. Dev. Agency.

[36] da Silva C.A., Santos S.O., Garcia C.A.B., de Pontes G.C., Wasserman J.C. (2020). Metals and arsenic in marine fish commercialized in the NE Brazil: Risk to human health. Hum. Ecol. Risk Assess. Int. J..

[37] Ormaza-González F.I., Ponce-Villao G.E., Pin-Hidalgo G.M. (2020). Low mercury, cadmium and lead concentrations in tuna products from the eastern Pacific. Heliyon.

[38] Bae S.J., Shin K.S., Park C., Baek K., Son S.Y., Sakong J. (2023). Risk assessment of heavy metals in tuna from Japanese restaurants in the Republic of Korea. Ann. Occup. Environ. Med..

[39] Núñez R., García M.Á., Alonso J., Melgar M.J. (2018). Arsenic, cadmium and lead in fresh and processed tuna marketed in Galicia (NW Spain): Risk assessment of dietary exposure. Sci. Total Environ..

[40] Koesmawati T.A., Arifin Z. (2015). Mercury and arsenic content in seafood samples from Jakarta Fishing Port, Indonesia. Mar. Res. Indones..

[41] Olmedo P., Pla A., Hernández A.F., Barbier F., Ayouni L., Gil F. (2013). Determination of toxic elements (mercury, cadmium, lead, tin and arsenic) in fish and shellfish samples. Risk assessment for the consumers. Environ. Int..

[42] Jinadasa B.K.K.K., Rameesha L.R.S., Edirisinghe E.M.R.K.B., Rathnayake R.M.U.S.K. (2010). Mercury, Cadmium and Lead Levels in Three Commercially Important Marine Fish Species of in Sri Lanka. Sri Lanka J. Aquat. Sci..

[43] Jinadasa B.K.K.K., Chathurika G.S., Jayasinghe G.D.T.M. (2018). Mercury and cadmium in swordfish and yellowfin tuna and health assessment for Sri Lankan consumers. Food Addit. Contam. Part B.

[44] Ruelas-Inzunza J., Šlejkovec Z., Mazej D., Fajon V., Horvat M., Ramos-Osuna M. (2018). Bioaccumulation of As, Hg and Se in tunas Thunnus albacares and Katsuwonus pelamis from the Eastern Pacific: Tissues distribution and As speciation. Environ. Sci. Pollut. Res..

[45] Medico O., Pompa C., Moscatelli S., Chiappinelli A., Carosielli L., Chiaravalle A.E. (2020). Lead, cadmium and mercury in canned and unprocessed tuna: Six-years monitoring survey, comparison with previous studies and recommended tolerable limits. J. Food Comp. Anal..

[46] Wong C., Roberts S.M., Saab I.N. (2022). Review of regulatory reference values and background levels for heavy metals in the human diet. Reg. Toxicol. Pharmacol..

[47] Parang H., Esmaeilbeigi M. (2022). Total mercury concentration in the muscle of four mostly consumed fish and associated human health risks for fishermen and non-fishermen families in the Anzali Wetland, Southern Caspian Sea. Reg. Stud. Mar. Sci..

